# Supplementary material for: Modeling glioblastoma heterogeneity as a dynamic network of cell states
Source: Mol Syst Biol. 2021 Sep 16;17(9):e10105. doi: 10.15252/msb.202010105 (PMC8444284; doi:10.15252/msb.202010105)
Supplement: Supplementary file 6 — Source Data for Figure 5 [file MSB-17-e10105-s004.zip › Figure5A_sourcedata/GSEA_3071/hallmarks_stateIII.GseaPreranked.1623742540963/HALLMARK_APICAL_SURFACE.html]

Details for gene set HALLMARK\_APICAL\_SURFACE[GSEA]

|  || Dataset | state33071 |
| Phenotype | NoPhenotypeAvailable |
| Upregulated in class | na\_neg |
| GeneSet | HALLMARK\_APICAL\_SURFACE |
| Enrichment Score (ES) | -0.34215328 |
| Normalized Enrichment Score (NES) | -1.2707725 |
| Nominal p-value | 0.19342604 |
| FDR q-value | 0.3613906 |
| FWER p-Value | 0.999 |
Table: GSEA Results Summary

  

Fig 1: Enrichment plot: HALLMARK\_APICAL\_SURFACE      
 Profile of the Running ES Score & Positions of GeneSet Members on the Rank Ordered List

  

| PROBE | GENE SYMBOL | GENE\_TITLE | RANK IN GENE LIST | RANK METRIC SCORE | RUNNING ES | CORE ENRICHMENT || 1 | HSPB1 |  |  | 4 | 0.650 | 0.0933 | No |
| 2 | THY1 |  |  | 472 | -0.253 | 0.0108 | No |
| 3 | AFAP1L2 |  |  | 557 | -0.257 | 0.0266 | No |
| 4 | AKAP7 |  |  | 718 | -0.265 | 0.0243 | No |
| 5 | EFNA5 |  |  | 1018 | -0.280 | -0.0114 | No |
| 6 | SRPX |  |  | 1747 | -0.322 | -0.1507 | No |
| 7 | BRCA1 |  |  | 1844 | -0.327 | -0.1278 | No |
| 8 | CROCC |  |  | 1907 | -0.330 | -0.0957 | No |
| 9 | PLAUR |  |  | 2181 | -0.347 | -0.1150 | No |
| 10 | ATP8B1 |  |  | 3071 | -0.428 | -0.2800 | Yes |
| 11 | GATA3 |  |  | 3260 | -0.453 | -0.2624 | Yes |
| 12 | GAS1 |  |  | 3494 | -0.505 | -0.2485 | Yes |
| 13 | DCBLD2 |  |  | 3543 | -0.525 | -0.1846 | Yes |
| 14 | SHROOM2 |  |  | 3552 | -0.527 | -0.1101 | Yes |
| 15 | SULF2 |  |  | 3776 | -0.708 | -0.0643 | Yes |
| 16 | APP |  |  | 3779 | -0.710 | 0.0383 | Yes |
Table: GSEA details [plain text format]

  

Fig 2: HALLMARK\_APICAL\_SURFACE: Random ES distribution      
 Gene set null distribution of ES for **HALLMARK\_APICAL\_SURFACE**
